# Psychological, social and biological determinants of ill health (pSoBid): Study Protocol of a population-based study

**DOI:** 10.1186/1471-2458-8-126

**Published:** 2008-04-21

**Authors:** Yoga N Velupillai, Chris J Packard, G David Batty, Vladimir Bezlyak, Harry Burns, Jonathan Cavanagh, Kevin Deans, Ian Ford, Agnes McGinty, Keith Millar, Naveed Sattar, Paul Shiels, Carol Tannahill

**Affiliations:** 1 Glasgow Centre for Population Health, Level 6, 39 St Vincent Place, Glasgow, G1 2ER, UK; 2 NHS Greater Glasgow and Clyde, Glasgow Royal Infirmary, Dept. Vascular Biochemistry, 4th Floor University Block, 10 Alexandra Parade, Glasgow, G31 2ER, UK; 3 MRC Social and Public Health Sciences Unit, 4 Lilybank Gardens, Glasgow, G12 8RZ, UK; 4 Robertson Centre for Biostatistics, University of Glasgow, Level 11, Boyd Orr Building, University Avenue, Glasgow, G12 8QQ, UK; 5 Scottish Executive, St. Andrew's House, Regent Road, Edinburgh, EH1 3DG, UK; 6 Section of Psychological Medicine, Faculty of Medicine – University of Glasgow, Gartnavel Royal Hospital, 1055 Great Western Road, Glasgow, G12 0XH, UK; 7 NHS Greater Glasgow and Clyde, Glasgow Royal Infirmary, Dept. of Clinical Biochemistry, Macewen Building, 84 Castle Street, Glasgow, G4 0SF, UK; 8 Rosehill, Munros Street, Alexandria, Dunbartonshire, G83 0PU6, UK; 9 University of Glasgow, Division of Cardiovascular and Medical Sciences based at Vascular Biochemistry, 4th Floor QEB, Glasgow Royal Infirmary, 10 Alexandra Parade, Glasgow, G31 2ER, UK; 10 University of Glasgow, Faculty of Medicine, University Dept. Surgery, Level 2, Q.E.B, Glasgow Royal Infirmary, 10 Alexandra Parade, G31 2ER, UK

## Abstract

**Background:**

Disadvantaged communities suffer higher levels of physical and mental ill health than more advantaged communities. The purpose of the present study was to examine the psychosocial, behavioural and biological determinants of ill health within population groups in Glasgow that differed in socioeconomic status and in their propensity to develop chronic disease especially coronary heart disease and Type 2 diabetes mellitus.

**Methods:**

Participants were selected at random from areas known to be at the extremes of the socioeconomic continuum in Glasgow. Within the categories of least deprived and most deprived, recruitment was stratified by sex and age to achieve an overall sample containing approximately equal numbers of males and females and an even distribution across the age categories 35–44, 45–54 and 55–64 years. Individuals were invited by letter to attend for assessment of their medical history, risk factor status, cognitive function and psychological profile, morbidity, and carotid intima-media thickness and plaque count as indices of atherosclerosis. Anonymised data on study subjects were collected from the General Practice Administration System for Scotland to analyse characteristics of participants and non-participants.

**Results:**

700 subjects were recruited. The response (active participants per 100 invitation letters) in the least deprived group was 35.1% and in the most deprived group was 20.3%. Lowest response was seen in young males (least deprived 22.4% and most deprived 14.1%).

**Conclusion:**

This cross-sectional study recruited the planned sample of subjects from least deprived and most deprived areas within Glasgow. As evident in other studies response differed between the most and least deprived areas. This study brought together researchers/academics from diverse disciplines to build a more sophisticated understanding of the determinants of health inequalities than can be achieved through unidisciplinary approaches. Future analyses will enable an understanding of the relationships between the different types of measure, and of the pathways that link poverty, biology, behaviour and psychology and lead to health inequalities.

## Background

Heart disease, diabetes, some cancers, rheumatoid arthritis and mental illness are examples of the burden of ill-health that is carried disproportionately by deprived communities[1]. Not only is the prevalence and incidence of disease higher in areas of deprivation but also the nature of the problem appears to be qualitatively different, and treatment less successful[2]. This inequality in disease risk can partially be explained by the higher prevalence of classical risk factors in deprived areas, but this explanation fails to account for the totality of the variations [3-5].

There are social gradients in a range of biological and psychosocial variables which indicate that living in a deprived environment may increase the propensity to develop chronic disease, through as yet unknown mechanisms[6,7]. A potential underlying cause of increased prevalence of disease is chronic inflammation. This has been observed to be more common in deprived than affluent populations, [8-12] linked to coronary heart disease[13,14], increased risk of type 2 diabetes[15,16] and other disorders[17], as well as cognitive dysfunction and altered psychological profile [18-21]. Atherosclerosis is now understood to be an inflammatory disorder with key components of the innate immune system being intimately involved in the initiation and progression of plaques on the artery wall, and in triggering an acute coronary event such as myocardial infarction[14]. The aetiology of diabetes appears also to involve activation of innate immunity with high levels of inflammatory biomarkers such as C reactive protein being associated with increased risk of developing the disorder[15]. Because of the aetiological links, some suggest that coronary heart disease and type 2 diabetes arise from a 'common soil'[22].

The cause of increased activation of the innate immune system in individuals from deprived populations is not clear. It may be linked to poor living and working conditions such as exposure to pathogens, or to increased levels of obesity. There is abundant evidence linking the accumulation of abdominal fat to raised levels of inflammatory cytokines in the plasma[23,24]. Further it is noteworthy that the relationship between body fat and inflammation is present even in children[25] suggesting that propensity to some adult chronic diseases may begin early in life. Abdominal obesity is believed to be a major precipitating factor in the development of insulin resistance and ultimately in the development of type diabetes[26]. For this reason the present 'epidemic' of obesity in many countries around the world is a significant concern for public health authorities and healthcare providers.

Chronic inflammation, central obesity and insulin resistance have been associated in population surveys and in experimental studies with impaired cognitive function and with an altered (negative) mental outlook. Depression appears to be more frequent in overweight individuals[27,28] and type 2 diabetes is recognised increasingly as a risk factor for accelerated cognitive decline in the elderly[29,30]. Prospective studies have shown inflammatory markers to predict cognitive decline in initially-healthy elderly subjects over follow-ups of between one and ten years[19,30], and that those of lower socio-economic status and poorer educational attainment are more vulnerable to such inflammation-related decline[31]. Recently investigators have reported that a further feature of central obesity/metabolic syndrome is an altered mental state associated with depression and "loss of control"[31,32]. It has also been recognised that depression is commonly found in subjects with CHD and is an important factor to overcome as part of recovery from a myocardial infarction [33-35].

These aetiological links need further exploration as potential explanations of the burden of physical and mental ill health in deprived communities. From a public health perspective it is important to establish if those who need to take on board messages advocating lifestyle change (weight loss, physical activity) are in a position affectively and intellectually to receive them. Equally, certain personality and other individual difference factors modify responses to stress and challenge, conferring both vulnerability and protection, and must be accounted for as moderating variables.

Glasgow is a particularly appropriate setting for a study of the effects of deprivation on ill-health because of the strong socioeconomic gradient within the conurbation, the fact that deprived communities make up a substantial proportion of the population and the associated variation in mortality and morbidity[7]. The present study is to determine the extent to which the syndrome of central obesity/chronic inflammation explains the socioeconomic gradient in vascular disease and whether the syndrome is associated with alterations in the mental state.

## Methods

### Aims and Hypotheses

The study for the most part was an exploratory pilot for a large scale investigation of the genotypic and phenotypic determinants of ill health in deprived communities The overall aim was to determine the extent to which the linked syndrome of central obesity/chronic inflammation explains the social gradient in vascular disease and whether the syndrome is associated with alterations in the mental state.

The hypotheses to be tested were summarised as follows in the study protocol:

"Socioeconomic gradients in health are influenced by adverse environmental conditions, work, relationships, community, knowledge and practice of health-promoting or health-damaging behaviours. Hormonal and metabolic responses to the above stressors, while protective in the short term, in the long term causes adverse changes (e.g. hyperplasia of visceral adipose tissue and central obesity) that leads to chronic disease (e.g. atherosclerosis). Further consequences are a heightened response to stress and a tendency towards depression and altered mental function".

### Research questions

The following questions were addressed:

1) Do deprived sections of the community display increased prevalence of features of a condition termed metabolic syndrome (i.e. central obesity and insulin resistance) and chronic inflammation compared to affluent sections?

2) Do deprived groups exhibit higher levels of serum endotoxin, revealing increased exposure to bacterial pathogens (as a result for example of damp housing) compared to affluent groups?

3) Do deprived groups differ from affluent ones in psychological profile (affective state and cognition) and to what extent can this be related to the presence of the central obesity/insulin resistance/chronic inflammation syndrome?

4) Is sub-clinical atherosclerosis (as detected by carotid ultrasound analysis) more prevalent in deprived groups? To what extent is the prevalence explained by classical risk factors (smoking, blood pressure, cholesterol) and to what extent is it related to the presence of metabolic syndrome?

In addition, we sought to ascertain the feasibility of a large scale population study by determining response rate, drop out rate, time taken by respondents to complete the questionnaires and the visits, any discomfort experienced by respondents to the various medical assessments, numbers volunteering for the Magnetic Resonance Imaging (MRI) Scan etc, and how the above were affected by age group, sex and deprivation category.

### Ethical approval and confidentiality

Ethical approval for the study was obtained from Glasgow Royal Infirmary Research Ethics committee. In all study records (electronic and paper) subjects were identified only by their study number. Information linking identity (name, address, general practitioners) to study number was held securely by the Glasgow Centre for Population Health[36] (GCPH; the coordinating centre). Only anonymised data were obtained from General Practice Administration System for Scotland[37] (GPASS) records on practice computers. The Health Board's Caldicott Guardian approved the study process and GPs with the approval of the ethics committee consented to the use of Community Health Index[38] and anonymised GPASS data.

The Health Information and Technology (HIT) section of the Greater Glasgow Health Board (GGHB) was responsible for sample selection and assignment of a study number to each subject (from 0001 to 3,600).

### Subjects

Selection was based on the Scottish Index for Multiple Deprivation[39] (SIMD) which identified the least and most deprived areas in the Glasgow conurbation area. Five general practitioners (GP) practices with the highest percentage of patients aged 35–64 years living in areas classified as being in the bottom 5% of SIMD [most deprived (MD)] were approached and all agreed to participate in the recruitment process. A further five practices with the highest percentage of patients aged 35–64 years living in areas classified as being in the top 20% of SIMD [least deprived (LD)] also agreed to participate.

HIT generated a target population of 21,672 people from the GP lists of these ten practices (Table 1). From this target population 12 groups of 300 each were selected according to strata defined by the combination of category, sex and age-group (35 to 44, 45 to 54, and 55 to 64 years) (Table 1) giving a total sampling frame of 3,600 subjects. As the study progressed, over-sampling of subjects from the most deprived group was required (due to the lower response rate) and the HIT section was approached to select randomly further potential subjects from the target population. GPs were able to exclude persons from the sample who had recently expired or who had a terminal illness. Due to the nature of the psychological questionnaires and cognitive assessment, only those who understood and spoke English were invited to participate in this pilot study. The eligibility of subjects was checked by GPs and Practice Managers before letters were sent.

**Table 1:** pSoBid target population by age and sex identified SIMD 2004

	Males				Females				Both Sexes
		
Number of subjects living in:	35–44 years	45–54 years	55–64 years	Total	35–44 years	45–54 years	55–64 years	Total	35–64 yrs
20% Least Deprived area*	2,124	2,169	2,024	6,317	2,278	2,335	2,074	6,687	13,004
5 % Most Deprived area*	1,931	1,482	949	4,362	1,849	1,366	1,091	4,306	8,668

Total	4,055	3,651	2,973	10,679	4,127	3,701	3,165	10,993	21,672

* The most deprived sample was drawn from the category 5% most deprived by SIMD. The least deprived sample was drawn from subjects living in areas classed by SIMD as in the 20% least deprived category

If the participant had had an illness which was likely to increase CRP levels acutely (e.g. urinary tract infection, upper respiratory tract infection, etc.) during the two weeks prior to his/her appointment this was recorded but assessments proceeded on the scheduled date.

### Recruitment Procedure

Invitation letters to selected subjects were sent in batches of 150 every two weeks. Accompanying the letter was a form for the subject to return (in a reply paid envelope) recording their contact details and indicating their willingness to consider participation. Subjects who agreed to receive further information about the study were sent the pSoBid participant information booklet[40]. If there was no response after two weeks, a reminder was sent. The Research Nurse contacted those who received the participant information booklet, and if after reading the information booklet they decided to participate in the study, they were invited to come for the first visit at their GP's clinic on a mutually agreed day and time (see Additional file 1 for flowchart). This process continued until approximately equal numbers for the 12 groups were recruited.

### Protocol

The study comprised two visits, each lasting about an hour and a half to two hours. Arrangements were made for taxi transfers to and from the participants' homes. Posters advertising the study were displayed in GP Clinics and also in local community centres and libraries. Two free telephone numbers were set up one in the coordinating centre and one in the Glasgow Royal Infirmary (GRI) where the research nurses were based.

At Visit 1 the study was explained to participants and informed consent obtained. The visit involved completion of lifestyle and psychology questionnaires, assessment of health status and measurement of blood pressure, pulse rate and indices of obesity (height, weight, hip, waist and mid thigh circumference). Lung function was measured by Forced Expiratory Volume in one second (FEV_1_) and Forced Vital Capacity (FVC). Questionnaires completed at this visit examined affective state and control/coping i.e. the General Health Questionnaire[41] (which has been used previously in this context by other research groups[31,42]), the Generalised Self-Efficacy Scale[43], the Sense of Coherence Scale[44] and Beck Hopelessness Scale[45]. An appointment was made for the second visit (in the morning and fasting) to be carried out at GRI on a date convenient to the participant.

At Visit 2, a fasting blood sample was taken to measure cholesterol, triglycerides, very low density lipoprotein (VLDL), low density lipoprotein (LDL) and high density lipoprotein (HDL), markers of diabetes and obesity (glucose, insulin, leptin and adiponectin), markers of inflammation and clotting [C-reactive protein (CRP); inerleukin-6, (IL6); fibrinogen, D dimer; tissue plasminogen activator (tPA) antigen], and markers of endothelial dysfunction [Intercellular Adhesion Molecule (ICAM); von Willebrand Factor (vWF)]. Then, after breakfast, participants completed further psychological and cognitive function tests, and underwent ultrasound assessment of carotid intima media thickness and plaque count. Previous research has shown an association between eating breakfast and mood and performance, with the effects due in part to experimental manipulation of the normal morning routine[46,47]. In this study as far as possible breakfast was provided according to an individual's normal routine (or abstinence, if relevant), so that any effects on performance and affective state would be those observed in real life.

Psychology questionnaires completed by the participants at Visit 2 provided indices of personality and individual differences in self-esteem. The personality factor of neuroticism is known significantly to affect emotional responsiveness and adjustment. Assessment involved self-completion of the Eysenck Personality Scales[48] and the Rosenberg Self-Esteem Scale[49]. Cognitive assessment involved the following main domains of cognition: executive function (tested by Trails Test[50] and Stroop Test[51]), memory (tested by Auditory Verbal Learning Test) and cognitive performance (estimated from the NART-2[52] which provided a proxy measure of "IQ"). Attention and speed of processing were tested by Choice Reaction Time[53].

Male participants were asked if they would be interested in participating in MRI scanning (Visit 3). From a total of 327 male participants, 140 volunteered, and 40 of these were randomly selected (stratified by age group and deprivation category). These scans will be completed by spring 2008.

Before each visit the participants were contacted by telephone on the previous day to confirm their attendance and to ensure that the taxi arrangements were in place. At the end of the study all participants were sent a letter thanking them for their participation in the study and were informed that they would all receive an executive summary of the study findings. After each visit participants were asked to complete a feedback form detailing their opinion of the study and their experiences.

### Lifestyle questionnaire

This questionnaire had 13 sections including basic demographic data, past and present health status, current medications, oral health, smoking history, alcohol intake, diet, physical activity levels, childhood situation, birth weight and place of birth, their parents' age and father's occupation, education levels, employment history and income levels.

### Carotid intima media thickness

Measurement of the intima media thickness of the carotid artery by high resolution ultrasound is now a widely accepted, non-invasive, surrogate measure of atherosclerosis and a reliable indicator of future risk of a major coronary event[54,55]. Carotid intima media thickness provides a suitable continuous outcome measure for atherosclerosis, enabling association studies to be performed on fewer numbers (i.e. hundreds of subjects compared to classical surveys using endpoints such as MI which require sample sizes in the thousands). Recent carotid intima media thickness studies have evaluated the extent to which sex differences in CHD are explained by central obesity, the relationship between degree of atherosclerosis (intima media thickness) and inflammation status (CRP levels)[55,56] and the relationship of periodontal disease to carotid intima media thickness[57]. Ultrasound examination of the carotid arteries also allows presence and number of plaques to be determined[58,59]. Carotid plaque count has previously been found to be a predictor of myocardial infarction[58] and stroke[60]. The carotid ultrasound examination lasted 20 to 30 minutes. Doppler velocity in right and left internal carotid arteries was recorded in order to identify any significant internal carotid artery stenosis. Images of the distal 1 cm of the common carotid artery, the carotid bulb and the proximal internal carotid artery were recorded on the left and right side, and intima-media thickness of the far wall of the artery determined using the software package Etrack. The number of carotid plaques at each of the six sites was determined using published procedures[58]. M-mode ultrasound of the distal common carotid artery was recorded to assess arterial stiffness. Reading of the scans was performed off-line by a reader who was blinded to the identity of the participants.

### GPASS Extraction Process

GPASS was used to evaluate the characteristics of those who were invited to participate in the study. Eight of the ten GP practices (four in the LD area, four in the MD area) selected for the study use GPASS to record their routine data. Anonymised data were collected on smoking status and current prescription for statins, aspirin, antihypertensives, antidepressants and anti-diabetic drugs as evidence of the prevalence of chronic disease. Data were collected separately for those who attended visit 1 (Group 1), those who declined to attend (Group 2) and non-respondents to the invitation (Group 3). Non-participants (Group 4) were defined as the combination of groups 2 and 3.

### Statistical analysis

Sample size in the LD and MD groups was estimated on the assumption that 90% would attend both visits and have CRP measured and that a maximum of 10% would not have good quality intima media thickness measurements. The power calculations were based on perceived clinically meaningful differences and assumed a 1.1 mg/L standard deviation for the natural logarithm of CRP measurements[61] and a 0.163 mm standard deviation for carotid intima media thickness[62]. Power calculations indicated that a sample size of 350 per group would provide 84% power to detect a 30% difference in mean CRP levels and 82% power to detect a 0.04 mm difference in mean carotid intima media thickness.

Categorical data are presented as counts and where appropriate as percentages. Where formal comparisons of percentages have been carried out, chi-squared tests have been used.

## Results

**Figure 1:**
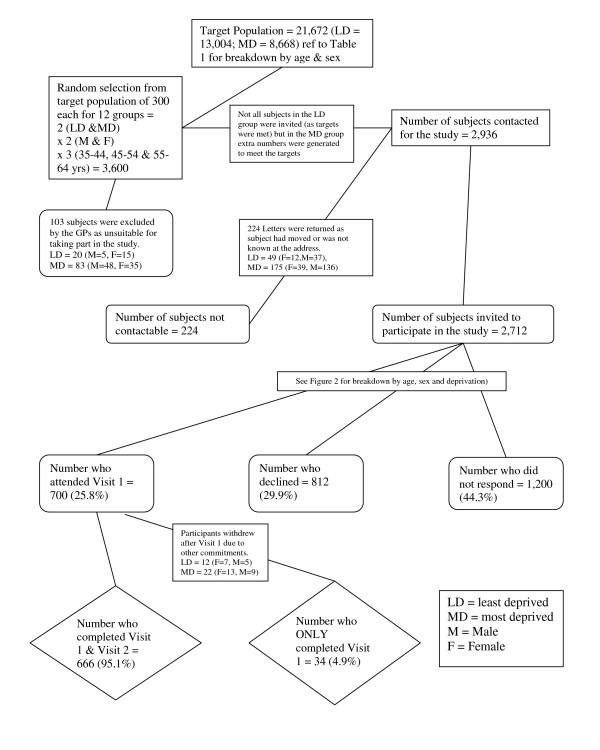
**Recruitment flowchart for the pSoBid study**. LD = Least Deprived; MD = Most Deprived. M = Male; F = Female.

From the sampling frame of 3600 subjects a total of 2,712 invitations were issued to recruit a cohort of 700 (25.8%) participants. Out of the 2,712 invitations sent, 812 (29.9%) people declined to participate and 1,200 (44.3%) did not respond (Figure 1). Data collection was completed in April 2007 and data quality was tested over the summer of 2007. For calculation of response rate the denominator used was the total number invited to participate in the study.

There were 224 people in the sample who were not contactable. Letters were returned by the postal services because the addressee had moved or was not known at that address (Figure 1). Males in the MD category were more often not contactable (12.4%) compared to the other subjects (6.6% LD males, 5% MD females and 2.4% LD females).

GPs removed 103 subjects from the sample as they felt that the subjects were not able to complete the study (house bound, too ill to participate, terminal illness or literacy problems). Eighty-three were from the MD subjects (35 females and 48 males) and 20 were from the LD subjects (15 females and 5 males).

### Response

**Figure 2:**
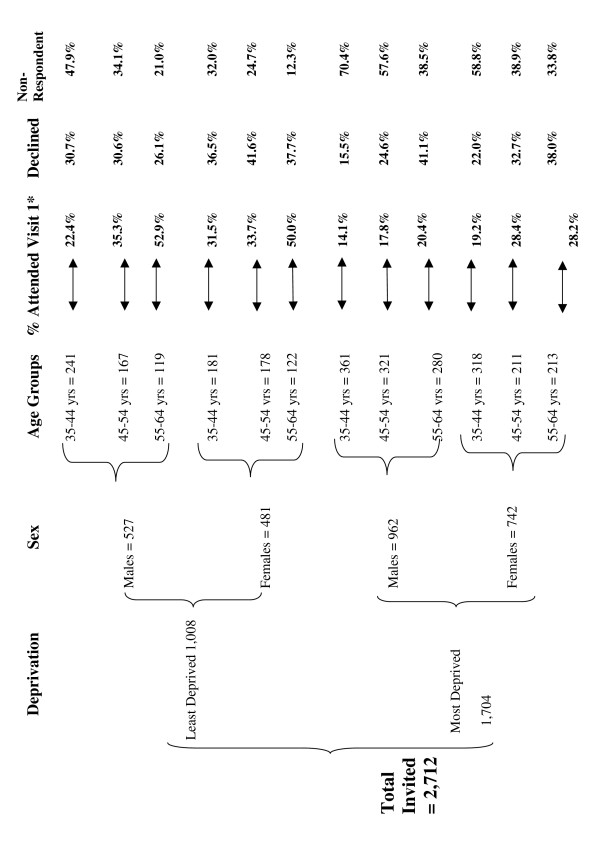
**Basic characteristics of subjects included in the pSoBid study by deprivation, sex and age**. * = Included in these % are 34 participants who ONLY completed Visit 1.

The number of letters sent in each of the 12 groups to recruit a target of 60 subjects was varied according to the group's response rate (Table 2). The highest number of letters (361) was sent to 35–44 year old males in the MD group (response rate = 14%); the fewest (119 and 122 respectively) were sent to 55–64 year old males and females in the LD participants (response rate 52.9% and 50% respectively – Figure 2). Although the initial target was 60 per group (720 in total), we stopped recruitment at 700 (in line with the power calculation) due to time constraints.

**Table 2:** Breakdown of pSoBid study subjects

**Sex/Depcat**	**Age Group**	**Completed Visit 1 & 2**	**Withdrew after Visit 1**	**Replied 'No'**	**Non-Respondents**	**Sent Letters**
**LD Female**	**35–44**	55 (30.4%)	2 (1%)	66 (36.5%)	58 (32.1%)	181 (100%)
**LD Female**	**45–54**	56 (31.5%)	4 (2.2%)	74 (41.6%)	44 (24.7%)	178 (100%)
**LD Female**	**55–64**	60 (49.2%)	1 (0.8%)	46 (37.7%)	15 (12.3%)	122 (100%)
**LD Female**	**Total**	**171 (35.6%)**	**7 (1.4%)**	**186 (38.7%)**	**117 (24.3%)**	**481 (100%)**
**LD Male**	**35–44**	52 (21.6%)	2 (0.8%)	74 (30.7%)	113 (46.9%)	241 (100%)
**LD Male**	**45–54**	58 (34.7%)	1 (0.6%)	51 (30.6%)	57 (34.1%)	167 (100%)
**LD Male**	**55–64**	61 (51.2%)	2 (1.7%)	31 (26.1%)	25 (21.0%)	119 (100%)
**LD Male**	**Total**	**171 (32.4%)**	**5 (1%)**	**156 (29.6%)**	**195 (37.0%)**	**527 (100%)**
***LD Participants***		***342 (33.9%)***	***12 (1.2%)***	***342 (33.9%)***	***312 (31%)***	***1008 (100%)***
**MD Female**	**35–44**	55 (17.3%)	6 (1.9%)	70 (22.0%)	187 (58.8%)	318 (100%)
**MD Female**	**45–54**	55 (26%)	5 (2.4%)	69 (32.7%)	82 (38.9%)	211 (100%)
**MD Female**	**55–64**	58 (27.2%)	2 (1%)	81 (38.0%)	72 (33.8%)	213 (100%)
**MD Female**	**Total**	**168 (22.6%)**	**13 (1.8%)**	**220 (29.6%)**	**341 (46.0%)**	**742 (100%)**
**MD Male**	**35–44**	49 (13.6%)	2 (0.5%)	56 (15.5%)	254 (70.4%)	361 (100%)
**MD Male**	**45–54**	53 (16.5%)	4 (1.3%)	79 (24.6%)	185 (57.6%)	321 (100%)
**MD Male**	**55–64**	54 (19.3%)	3 (1.1%)	115 (41.0%)	108 (38.6%)	280 (100%)
**MD Male**	**Total**	**156 (16.2%)**	**9 (0.9%)**	**250 (26.0%)**	**547 (56.9%)**	**962 (100%)**
***MD Participants***		***324 (19.0%)***	***22 (1.3%)***	***470 (27.6%)***	***888 (52.1%)***	***1704 (100%)***
**GRAND**	**TOTAL**	**666 (24.6%)**	**34 (1.3%)**	**812 (29.9%)**	**1,200 (44.2%)**	**2712 (100%)**

**LD = Least Deprived**.

MD = Most Deprived

The total response rates were calculated by combining those who completed Visit 1 & 2 (column 3) with those who withdrew after Visit 1 (column 4).

Of the 700 subjects who participated in the study only 34 (4.9%) did not complete both visits. Of these, 12 were 35–44 years old; 14 were 45–54 years and 8 were 55–64 years (Table 2).

There were 171 male and 171 female participants in the LD group and 168 females and 156 males in the MD group (Table 2). The response rate was 33.9% for LD and 19.0% for MD participants, and response rate by age group was 31.7% in 35–44 year olds, 33.3% in 45–54 year olds and 35% in 55–64 year olds.

A total of 812 subjects (Table 2) in the sample declined (replied NO to the invitation). There were more females who declined (LD = 38.7% and MD = 29.6%) than males (LD = 29.6% and MD = 26.0%). In the MD group (35–44 year olds were less likely to respond than were 55–64 year olds, but this age difference was not seen in the LD subjects.

A total of 1,200 people in the sample did not respond to the letter; the non-response was 52.1% for MD and 30.9% for LD subjects. More males did not respond (LD = 37% and MD = 56.9%) compared to females (LD = 24.3% and MD = 46%). There was an age difference for both males and females (non-response rate higher for 35–44 compared to 55–64 year olds) in both MD and LD subjects.

## Discussion

**Figure 3:**
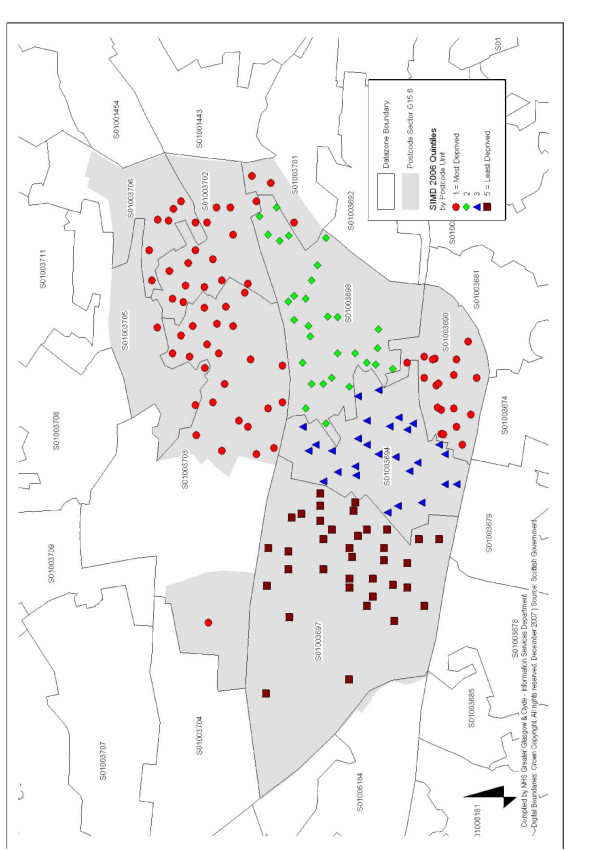
**Map showing Postcode Sector G15 6 by DECPAT and SIMD Data Zones**. SIMD = Scottish Index for Multiple Deprivation.

The study was successful in recruiting subjects of the desired sex and age profile from the most and least deprived areas of Glasgow. Data zones in SIMD were the preferred choice for our sampling process because each covers a smaller area and population (750–1,000) than the Postcode sectors which were used in a previous, well used deprivation classification (DEPCAT)[63]. This is illustrated in Figure 3 which shows the Postcode sector G15 6 (DEPCAT 6) and the same area by data zones (SIMD quintiles 1–5). The G15 6 area has nine data zone areas which have clear boundaries between the various quintiles in the SIMD and consists of least and most deprived areas. The Information Services Division (ISD) Scotland has recommended that routine and in-depth NHS Board level analyses from 1997 onwards use SIMD measure of deprivation[64].

In recruiting for the present study we found similar patterns of response to those observed in earlier surveys. In the Scottish Health Survey of 2003 (SHS 2003) from the co-operating households, response was lowest among those aged 16–24. Among men, response was highest among those aged 65 plus, while among women those aged over 24 gave a consistently high response rate[65]. In SHS 2003, men aged below 35 years were slightly under-represented at both interview and nurse visit relative to their proportions in the population while men aged 55 and over were slightly over-represented. Women aged below 25 years were under-represented at both stages, while women aged 45–74 were overrepresented[65].

The availability of the GPASS information allowed us to examine at least some potential bias inherent in our recruitment strategy. Of particular concern was the possibility that, of those invited, the 'worried well' and 'healthy deprived' would preferentially volunteer, so minimising potential differences between the least and most deprived communities.

A further methodological concern was the impact on participation of the number of questionnaires, investigations and the prospect of having to attend two visits of 1 1/2 hours each for this study. Only 34 participants failed to complete both visits and the full set of evaluations. Participants stated on a feedback questionnaire that they were highly satisfied with the study process and some saw it as an opportunity to have a complete medical check-up

### Strengths and Limitations

The study achieved its recruitment objectives in terms of the sample size and the nature of the recruits. The depth and range of the evaluations performed will provide important information concerning the relationships between deprivation, obesity, inflammation, atherosclerosis and mental outlook. This will enable us to address the hypothesis that the increased prevalence of coronary heart disease, type 2 diabetes and negative mental outlook in a deprived population is attributable in large part to an increased frequency of chronic inflammation, endothelial dysfunction and insulin resistance linked to the more challenging social environment.

There are limitations to the design of pSoBid[66]. Since the sample was stratified by age and sex, it is not a true representation of the general population; further, there is bias due to the variation in response rate. The sample was selected from the extremes of deprivation so as to maximise any observed differences and, therefore, provides no information about population gradients. The cross-sectional nature of the study means that it will not be possible to identify causal pathways or the temporal relationship between variables.

That said, the breadth and depth of data collected, linkage to NHS records, and the population-based nature of pSoBid make it an important resource, now and in prospect, for building understanding about the mechanisms that help to explain deprivation-related ill-health.

## Conclusion

The multidisciplinary approach employed in this study will enable a more holistic understanding of the diverse characteristics of individuals who reside in affluent and deprived communities and their influence on health and health inequalities. This study also illustrates the willingness of subjects to volunteer for a variety of investigations involving psychological, behavioural, sociological and medical questions and tests including blood analysis. As in other studies it was easier to enrol females than males, older compared to younger people, and the more affluent participants. Linkage to medical records allowed comparison of the health characteristics of participants and non-participants, yielding an insight into aspects of volunteer bias in studies of this type. This study also brought together researchers/academics from diverse disciplines to build a more sophisticated understanding of the determinants of health inequalities than can be achieved through unidisciplinary approaches. Future analyses will enable an understanding of the relationships between the different types of measure, and of the pathways that link poverty, biology, behaviour and psychology and lead to health inequalities.

This article has outlined the study background, design and recruitment. The findings from this study will be presented in future articles.

## Competing interests

The author(s) declares that they have no competing interests.

## Authors' contributions

YV, CJP, DGB, HB, JC, KD, IF, KM, NS, PS and CT contributed equally to conception, design and final approval of the version to be published. YV, CJP and CT have been involved in drafting the manuscript and revising it critically for important intellectual content. VB performed the statistical analysis. AM supervised the recruitment of subjects and data collection.

## Pre-publication history

The pre-publication history for this paper can be accessed here:



## Supplementary Material

Additional file 1 Flow chart for subjects in pSoBid. Click here for file
