# Supplementary material for: Psychological, social and biological determinants of ill health (pSoBid): Study Protocol of a population-based study
Source: BMC Public Health. 2008 Apr 21;8:126. doi: 10.1186/1471-2458-8-126 (PMC2386810; doi:10.1186/1471-2458-8-126)
Supplement: Additional file 1 — Flow chart for subjects in pSoBid. [file 1471-2458-8-126-S1.doc]

Flow chart for subjects in pSoBid

| GP practices in the 20% Least & 20% Most deprived data zones in GGHB area were identified |
| --- |

| Five GP practices were selected in the 20% Least and 5% Most deprived areas in GGHB |
| --- |

| Subjects were randomly selected from GP lists to create a sampling frame |
| --- |

| Lists were generated for each GP Practice to exclude persons who had recently expired or who had a terminal illness |
| --- |

| Those eliminated by this process were replaced by randomly selecting from above sampling frame |
| --- |

| First letter was sent out with a reply form stating if willing to participate in the study |
| --- |

| Subject replied YES |  |  | Subject replied NO |
| --- | --- | --- | --- |

| Information sheet/pamphlet sent out and after 2 weeks contact was made to arrange for 1^st^ visit . |
| --- |

| No reply from subject after 2 weeks |
| --- |

| Reminder letter sent after 2 weeks with reply form |
| --- |

| Subject replied YES |  |  |  |  | Subject replied NO |
| --- | --- | --- | --- | --- | --- |

| 2^nd^ visit in GRI within 10-14 days in the morning (Fasting) |
| --- |

| NON Respondents |
| --- |

| *Request GP for anonymised characteristics* |
| --- |

If not known at the address or NA then we selected a new participant.
